# Adult Patient With Neuroblastoma Presenting as Acute Leukemia

**DOI:** 10.7759/cureus.27769

**Published:** 2022-08-08

**Authors:** Yu-Wei Lin, Yu-Hsin Hsu, Ming-Yuan Lee

**Affiliations:** 1 Department of Pathology and Laboratory Medicine, Koo Foundation Sun Yat-Sen Cancer Center, Taipei, TWN

**Keywords:** immunohistochemistry, differential diagnosis, cytomorphology, small blue round cell tumor, neuroblastoma

## Abstract

Neuroblastoma (NB) is the most common extra-cranial cancer of early childhood and rarely occurs in adults. The clinical symptoms of NB can be diverse. We discuss a rare case of an adult NB presenting as acute leukemia. A 45-year-old woman presented with persistent hip pain, weight loss, anemia, and incidental fever for several months. Imaging studies showed diffuse bone marrow (BM) uptake and hypermetabolic lesions involving the left adrenal gland, bilateral axillary nodes, and left lateral aspect of the abdomen. Her 24-hour urine catecholamines were within the normal range. On the peripheral blood film, blast-like cells were noted, occupying approximately 2% of leukocytes. The BM imprints showed infiltration of blast-like cells with convoluted nuclei and scant cytoplasm in more than 85% of the total nucleated cells. Acute leukemia was initially suspected based on morphology. Blast-like cells were negative for myeloperoxidase, combined esterase, periodic acid-Schiff, CD45RB, CD68, and CD138. In a further study, these cells were positive for CD56, synaptophysin (SYN), and CD99 with negativity for desmin, myogenin, NKX-2.2, CD31, cytokeratin (AE1/AE3), Melan-A, ERG, S-100, and SALL4. Morphologically similar neoplastic cells in axillary node biopsy were positive for CD56, chromogranin A, SYN, and neurofilament, but negative for GFAP, CD246, and vimentin. Based on laboratory, pathological, and imaging studies, metastatic NB with BM and multifocal involvement was diagnosed. The differential diagnosis of metastatic small blue round cell tumors should be considered for adult patients with circulating blast-like cells, and an accurate diagnosis would enable the patient to receive appropriate and timely treatment.

## Introduction

Neuroblastoma (NB) is an embryonal cancer arising from peripheral sympathetic nervous system and the most common extra-cranial cancer of early childhood [1]. NB affects one in 8000 live birth and represents 6-10% of all childhood tumors [2]. It occurs rarely in adults and commonly affects adults with age in the fifth to sixth decades [2]. The incidence rate of NB for patients over 30 years old is 0.2 cases per million inhabitants per year and its incidence becomes increasingly scarce in the elderly population [3].

NB commonly occurs in the adrenal medulla, neck, chest, and pelvis [4]. Metastatic NB commonly involves regional lymph nodes, bone marrow (BM), cortex, and liver [5]. Since NB may develop throughout the sympathetic nervous system, the clinical symptoms can be diverse depending on the location of the tumor [5]. We discuss a rare case of an adult NB involving multiple sites presenting as acute leukemia.

## Case presentation

**Figure 1:**
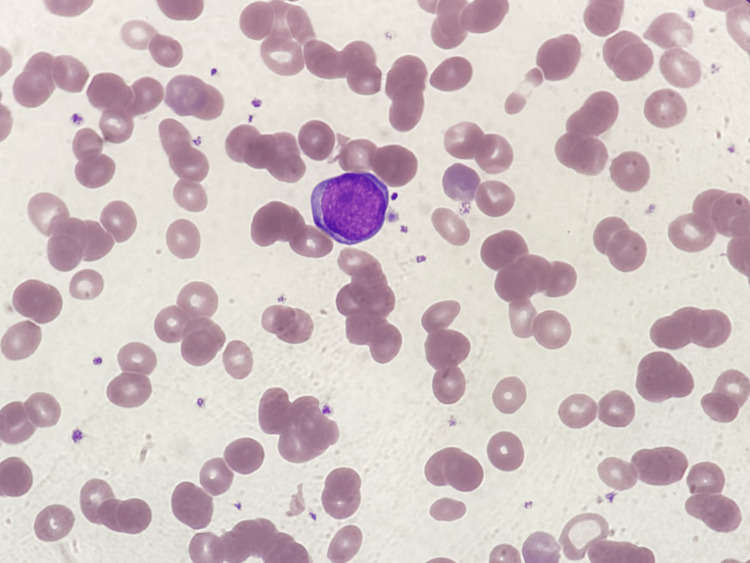
A blast-like cell on peripheral blood smear Blast-like cells occupying about 2% of leukocytes were found on peripheral blood smear and presented with approximately four times the size of an erythrocyte and deeply blue cytoplasm (Wright-Giemsa stain, 1000X).

A 45-year-old woman presented with persistent hip pain, weight loss, anemia, and incidental fever for several months. Imaging studies showed mild splenomegaly, diffuse BM uptake, and hypermetabolic lesions involving the left adrenal gland, bilateral axillary nodes, and left aspect of the lateral abdomen. Her 24-hour urine vanillylmandelic acid and catecholamines, including epinephrine, norepinephrine, and dopamine, were within the normal range. Laboratory tests were notable for elevated serum lactate dehydrogenase (LDH) and elevated serum ferritin. A complete blood count with differential showed microcytic anemia, and a peripheral blood smear revealed a few blast-like cells occupying approximately 2% of leukocytes (Table 1, Figure 1).

**Table 1:** Laboratory results of our case WBC, white blood cell count; RBC, red blood cell count; MCV, mean corpuscular volume; PLT, platelet count; LDH, lactate dehydrogenase; VMA, vanillylmandelic acid; EPI, epinephrine; NE, norepinephrine; DA, dopamine.

Test	Result	Reference value
WBC (x10^3^/µL)	4.26	3.2-9.0 for women
RBC (x10^6^/µL)	2.07	4.0-5.10 for women
Hemoglobin (g/dL)	5.8	12.0-15.5 for women
Hematocrit (%)	17.5	36-46 for women
MCV (fL)	84.5	85.0-101.0
PLT (x10^3^/µL)	252	150-400
LDH (U/L)	1537	106-211
Ferritin (ng/mL)	1350	13-150 for women
24-hour urine VMA (mg/day)	2.99	1.00-7.50
24-hour urine EPI (µg/day)	<3.0	<27.0
24-hour urine NE (µg/day)	32.5	<97.0
24-hour urine DA (µg/day)	203.6	<500.0

**Figure 2:**
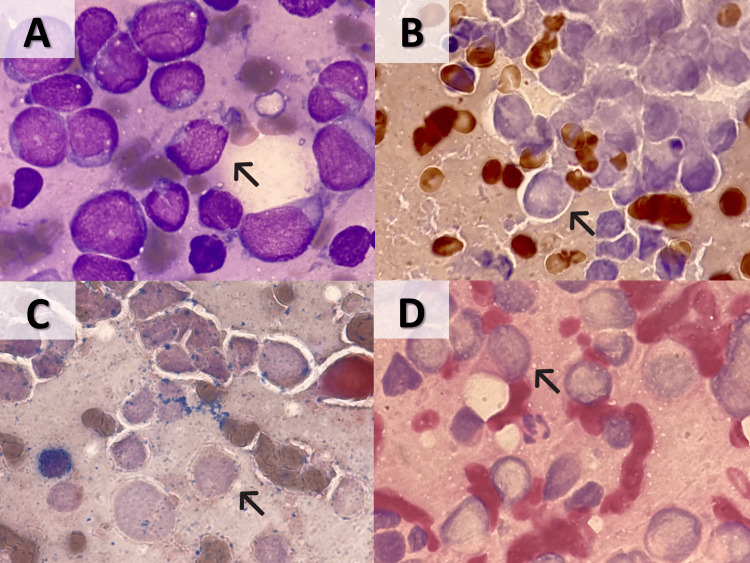
Morphologic and cytochemical features of BM imprints (A) Blast-like cells with convoluted nuclei and scant cytoplasm infiltrated the BM in more than 85% of the total nucleated cells (Wright-Giemsa stain, 1000X); (B) negative MPO stain (1000X); (C) negative CES stain (1000X); (D) negative PAS stain (1000X). BM, bone marrow; MPO, myeloperoxidase; CES, combined esterase; PAS, periodic acid-Schiff.

**Figure 3:**
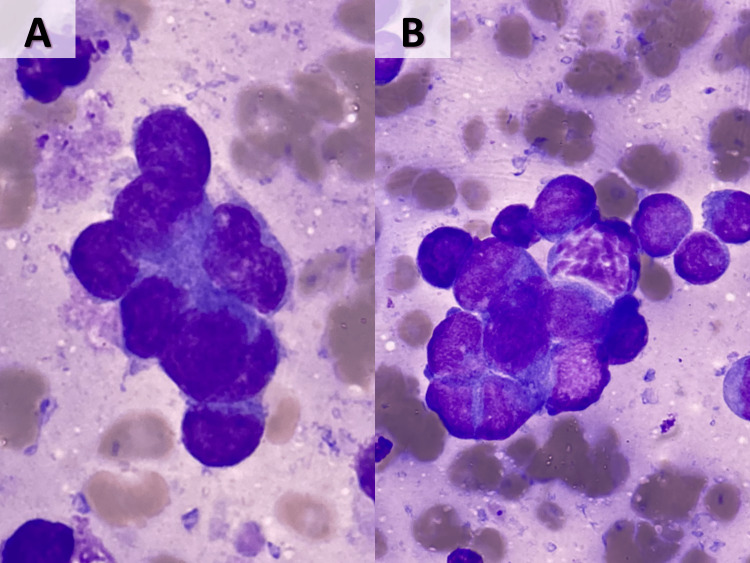
Homer-Wright rosettes on BM imprints (A) Homer-Wright rosettes were scarcely found on the BM imprint smear (Wright-Giemsa stain, 500X); (B) a Homer-Wright rosette with cell mitosis (Wright-Giemsa stain, 500X). BM, bone marrow.

BM examination and fine-needle aspiration at the enlarged axillary node were performed. The BM imprints showed infiltration of blast-like cells in more than 85% of the total nucleated cells with markedly decreased normal hematopoietic components (Figure 2A). Based on morphology only, acute leukemia was initially suspected. However, these blast-like cells were negative for myeloperoxidase (MPO), combined esterase, and periodic acid-Schiff (Figures 2B-2D). Most of the cells presented as a single cell, whereas the minority of the cells were concentrically arranged with fibrillar extension and formed so-called Homer-Wright rosettes (Figures 3A, 3B).

**Figure 4:**
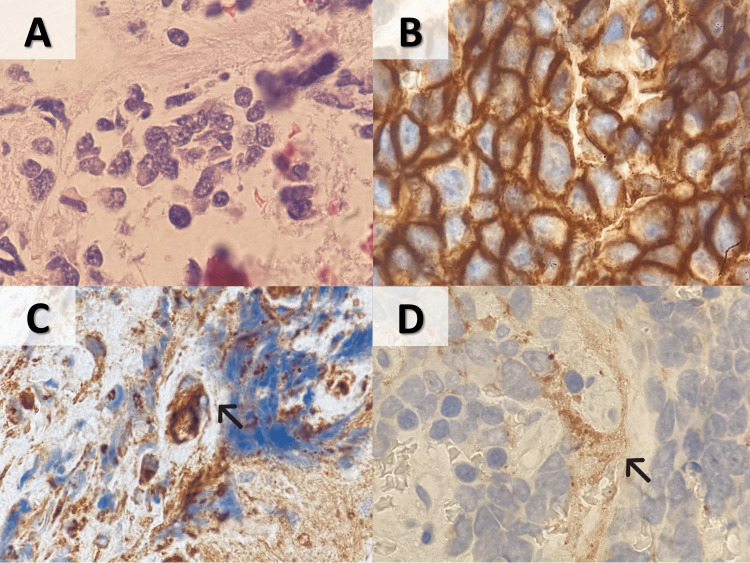
Morphologic and immunohistochemical features of neoplastic cells in bone marrow biopsy (A) Cells with hyperchromatic nuclei and scant cytoplasm (H&E stain, 1000X); (B) positive membranous staining for CD56 (1000X); (C) positive cytoplasmic staining for CD99 (1000X); (D) focal weak staining for SYN (1000X). H&E, hematoxylin and eosin; SYN, synaptophysin.

Morphologically similar neoplastic cells with hyperchromatic nuclei and scant cytoplasm were seen in BM biopsy tissue (Figure 4A). These cells were negative for hematologic markers (CD45RB, CD68, and CD138), making hematological malignancies less likely. Therefore, small blue round cell tumors (SBRCTs) were considered. On further study, these cells were positive for neural-related markers, including CD56, CD99, and synaptophysin (SYN) (Figures 4B-4D), with negativity for rhabdomyosarcoma (RMS) markers (desmin and myogenin), Ewing’s sarcoma (ES) marker (NKX-2.2), and other immunomarkers (CD31, cytokeratin (AE1/AE3), Melan-A, ERG, S-100, and SALL4).

**Figure 5:**
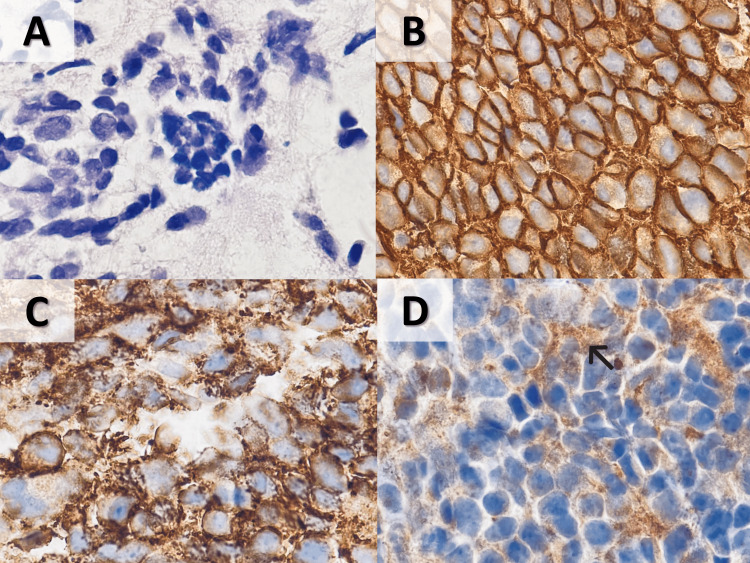
Morphologic and immunohistochemical features of neoplastic cells in axillary node biopsy (A) Small blue round cells (H&E stain, 1000X); (B) positive membranous staining for CD56 (1000X); (C) positive cytoplasmic staining for CGA (1000X); (D) positive cytoplasmic staining for SYN (1000X). H&E, hematoxylin and eosin; CGA, chromogranin A; SYN, synaptophysin.

**Figure 6:**
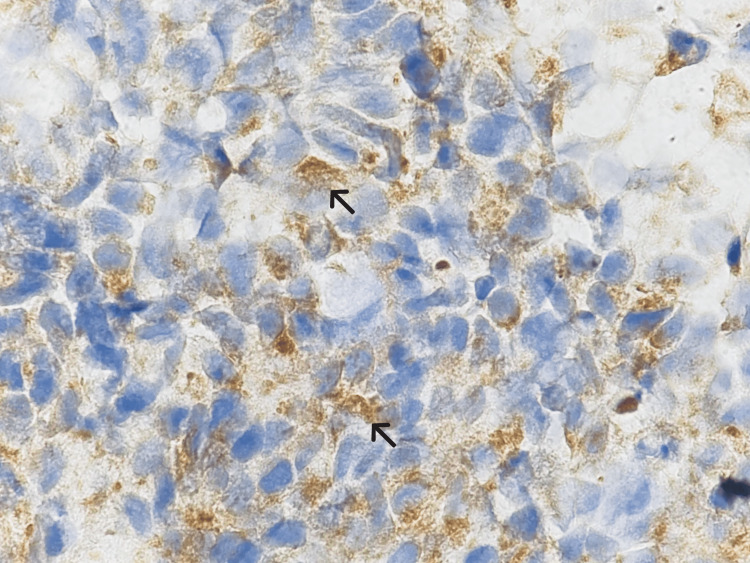
Positive expression of neurofilament protein in neuroblasts Neurofilament antibody was applied for confirmation of neuroblasts (1000X).

Morphologically similar small cells with hyperchromatic nuclei were seen in a biopsy from an enlarged axillary lymph node as well (Figure 5A). These neoplastic cells were positive for neural-related markers, including CD56, chromogranin A (CGA), SYN, and neurofilament (Figures 5B-5D, 6) but negative for GFAP, CD246, and Vimentin. These findings were consistent with neuroblasts.

Based on the laboratory test results, pathological findings, and imaging studies, metastatic NB with BM and multifocal involvement was diagnosed. The patient received radiation therapy at the left adrenal gland and adjuvant high-dose chemotherapy with autologous peripheral blood stem cell transplantation. She has kept follow-up appointments with the hematologist since then.

## Discussion

We presented a rare case of adult NB with the clinical manifestations mimicking acute leukemia. Our case had life-threatening anemia with circulating blast-like cells, and BM imprints revealed infiltration of these neoplastic cells in more than 85% of the total nucleated cells. Since the preliminary immunoprofiles were not compatible with a hematopoietic neoplasm, the diagnosis of SBRCT was taken into account. SBRCTs consist of a heterogeneous group of malignant neoplasms characterized by a monotonous population of poorly differentiated tumor cells with relatively small-sized, hyperchromatic nuclei, and scant cytoplasm [6].

The most common SBRCTs include non-Hodgkin’s lymphoma, NB, ES/primitive neuroectodermal tumor (PNET), RMS, and small cell neuroendocrine carcinoma (SNEC) [2]. The similar cytomorphology of SBRCTs makes it essential to rely on immunoprofiles to yield an accurate diagnosis. Immunohistochemical markers and histological features of SBRCTs included in our differential diagnoses are shown in Tables 2, 3. Hematopoietic neoplasms, RMS, SNEC, ES/PNET, a small cell variant of poorly differentiated squamous cell carcinoma, and other metastatic tumors, including melanoma, endothelial tumors, germ cell tumors, and central nervous system tumor, were excluded due to negative results for MPO, CD45RB, cytokeratin (AE1/AE3), and corresponding immunomarkers [2,7]. The positive reactivities for CD56, CGA, SYN, and neurofilament were most consistent with a diagnosis of metastatic NB.

**Table 2:** Comparisons of histological features for common SBRCTs and our case P, almost positive expression; N, almost negative expression; S, sometimes positive; NHL, non-Hodgkin’s lymphoma; RMS, rhabdomyosarcoma; ES, Ewing’s sarcoma; PNET, primitive neuroectodermal tumor; SNEC, small cell neuroendocrine carcinoma; NB, neuroblastoma; SBRCT, small blue round cell tumor. Based on data from Refs. [2,7-11].

	NHL (unclassified)	RMS	ES/PNET	SNEC	NB	NB (our case)
Pattern	Nodular, diffuse	Sheets, alveolar	Sheets, nests	Sheets	Diffuse, lobular	Diffuse
Morphology	Polymorphous, small to large cells with cleaved nuclei or blastoid cells	Round, strap and spindled cells	Medium and round cells with glycogen in cytoplasm (PAS stain positive)	Small cells with oval to spindled nuclei and scant cytoplasm	Small, round, and poorly differentiated cells with scant cytoplasm	Small to medium sized, poorly differentiated cells with scant cytoplasm
Neurofibrillary matrix	N	N	N	N	P	P
True rosettes or Homer-Wright rosettes	N	N	S	S	P	P

**Table 3:** Comparisons of IHC markers for common SBRCTs and our case P, almost positive expression; N, almost negative expression; S, sometimes positive; R, rarely positive; N/A, not applicable; NHL, non-Hodgkin’s lymphoma; ES, Ewing’s sarcoma; PNET, primitive neuroectodermal tumor; RMS, rhabdomyosarcoma; NB, neuroblastoma; IHC, Immunohistochemical; CGA, chromogranin A; SBRCT, small blue round cell tumor; SNEC, small cell neuroendocrine carcinoma. Based on data from Refs. [2,7-14].

	B-cell NHL	T/NK-cell NHL	RMS	ES/PNET	SNEC	NB	NB (our case)
CD45RB	P	P	N	N	N	N	N
CD20	P	N	N	N	N	N	N
CD56	R	P	P	R	P	P	P
CD99	R	R	R	P	N	N	P
SYN	N	N	R	S	P	P	P
Desmin	N	N	P	N	N/A	N/A	N
Myogenin	N	N	P	N	N	N	N
NKX-2.2	N/A	N/A	N/A	P	N/A	N/A	N
Cytokeratin (AE1/AE3)	N	N	S	R	P	R	N
S-100	N	N	R	S	R	P (sustentacular only)	N
CGA	N	N	R	R	P	S	P
GFAP	N	N	N	R	N	P (sustentacular only)	N
Vimentin	S	S	P	P	S	R	N
Neurofilament	N/A	N/A	N/A	N/A	N/A	P	P

The rarity of adult NB makes it a valuable opportunity to appreciate the complexity of the disease. The biological characteristics of NB may be different between young patients and adult counterparts. First of all, elevated urinary catecholamine is known as one of the features of NB. However, our case showed no elevation of catecholamines in her 24-hour urine as reported previously [8]. Furthermore, immune expression of NB might vary from adult cases to pediatric cases. A high level of CD246 or anaplastic lymphoma kinase protein has been detected in fetal neuroblasts [15]. There was an aberrantly negative expression of CD246 in our case. Finally, the prognosis of the disease shows a noticeable difference between adults and infants. For infant patients with NB, five-year survival is near 85%; in contrast, the overall survival rate at five years is 36% for adults. Adult patients, up to one-third, are diagnosed with metastasis [16].

Based on the International Neuroblastoma Staging System (INSS), our case with distant metastatic disease belongs to INSS stage IV, which indicates the worst outcome of all the other stages. Pediatric patients with NB can receive standard treatment with optimal risk stratification, while there are still no standard therapies for adult NB patients [4,16,17]. Though chromosomal and molecular studies, such as *MYCN* amplification, were not applied for risk assessment in our case, other laboratory findings including serum LDH and ferritin may be indicators of poor prognosis [17,18]. 

## Conclusions

The rarity and diverse symptoms of adult NB may lengthen the diagnostic process for these patients. For adults with circulating blast-like cells, the differential diagnosis of metastatic SBRCTs should be considered. Since we could not distinguish neuroblasts from hematological malignant cells by morphology, CD56, CGA, SYN, and neurofilament are essential immunomarkers for the differential diagnosis of NB. High suspicion and application of proper ancillary tests would allow accurate diagnosis and timely treatment.
